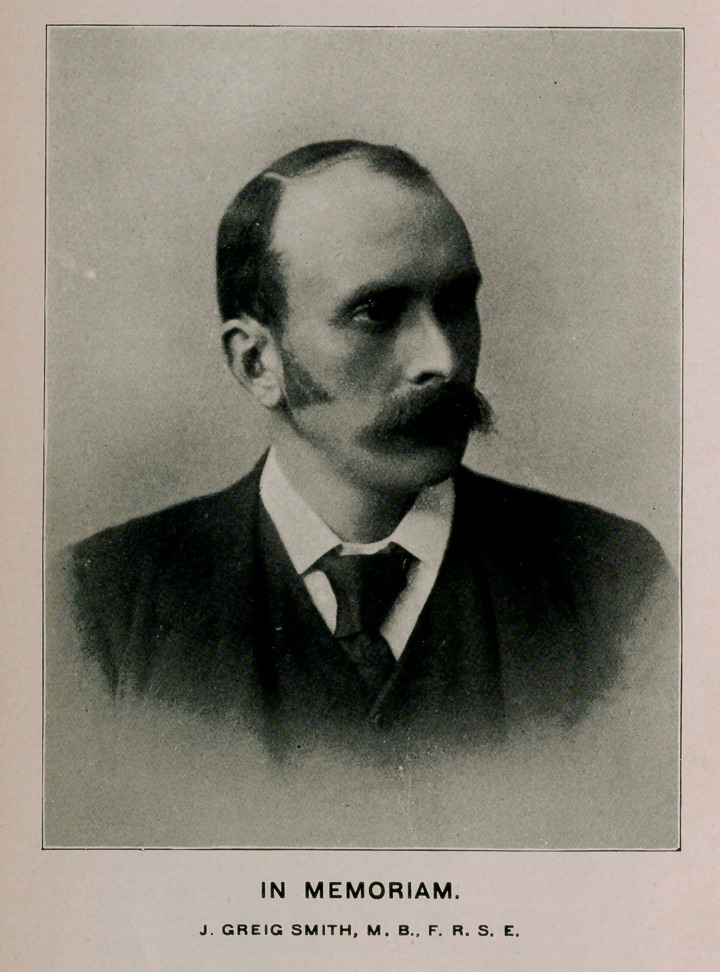# In Memorium

**Published:** 1898-01

**Authors:** 


### IN MEMORIAM.

J. GREIG SMITH, M. B., F. R. S. E.

FROM A RECENT PHOTOGRAPH.

*See page 937, Vol. XXXVI.*

## Figures and Tables

**Figure f1:**